# Prospective study on time-to-tertiary care in alcohol-associated hepatitis: space–time coordinates as prognostic tool and therapeutic target

**DOI:** 10.1093/alcalc/agae092

**Published:** 2025-01-20

**Authors:** Ľubomír Skladaný, Daniela Žilinčanová, Natália Kubánek, Svetlana Adamcová Selčanová, Daniel Havaj, Lukáš Laffers, Michal Žilinčan, Alvi H Islam, Juan Pablo Arab, Tomáš Koller

**Affiliations:** Department of Hepatology, Gastroenterology, and Transplantation (HEGITO), 2nd Department of Internal Medicine, Slovak Medical University Faculty of Medicine, F. D. Roosevelt Hospital, Námestie L. Svobodu 1, 974 01 Banská Bystrica, Slovakia; Department of Hepatology, Gastroenterology, and Transplantation (HEGITO), 2nd Department of Internal Medicine, Slovak Medical University Faculty of Medicine, F. D. Roosevelt Hospital, Námestie L. Svobodu 1, 974 01 Banská Bystrica, Slovakia; Department of Hepatology, Gastroenterology, and Transplantation (HEGITO), 2nd Department of Internal Medicine, Slovak Medical University Faculty of Medicine, F. D. Roosevelt Hospital, Námestie L. Svobodu 1, 974 01 Banská Bystrica, Slovakia; Department of Hepatology, Gastroenterology, and Transplantation (HEGITO), 2nd Department of Internal Medicine, Slovak Medical University Faculty of Medicine, F. D. Roosevelt Hospital, Námestie L. Svobodu 1, 974 01 Banská Bystrica, Slovakia; Department of Hepatology, Gastroenterology, and Transplantation (HEGITO), 2nd Department of Internal Medicine, Slovak Medical University Faculty of Medicine, F. D. Roosevelt Hospital, Námestie L. Svobodu 1, 974 01 Banská Bystrica, Slovakia; Department of Mathematics, Faculty of Natural Sciences, Matej Bel University, Tajovského 40, 974 09, Banská Bystrica, Slovakia; Department of Radiology, F.D. Roosevelt Hospital, Námestie L. Svobodu 1, 974 01, Banská Bystrica, Slovakia; Division of Gastroenterology, Department of Medicine, Schulich School of Medicine, Western University & London Health Sciences Centre, London, Rm. B0-692F, St. Joseph's Health Care, Ontario, Canada; Division of Gastroenterology, Department of Medicine, Schulich School of Medicine, Western University & London Health Sciences Centre, London, Rm. B0-692F, St. Joseph's Health Care, Ontario, Canada; Department of Epidemiology and Biostatistics, Schulich School of Medicine, Western University, London, 1465 Richmond Street, Ontario, Canada; Departamento de Gastroenterologia, Escuela de Medicina, Pontificia Universidad Catolica de Chile, Libertador Bernando O'Higgins Avenue 340, Santiago, Chile; Subdivision of Gastroenterology and Hepatology, 5th Department of Internal Medicine, Comenius University Faculty of Medicine, University Hospital Bratislava, Ružinovská 6, 826 06, Bratislava, Slovakia

**Keywords:** alcohol-associated hepatitis, survival, time, prognosis, tertiary care, secondary care, bundle of care, advanced chronic liver disease, cirrhosis, acute decompensation, trigger

## Abstract

**Background and aims:**

Alcohol-associated hepatitis (AH) frequently triggers acute decompensation (AD) in cirrhosis, with severe AH linked to high short-term mortality, especially in acute-on-chronic liver failure. Current corticosteroid treatments have limited efficacy, highlighting the need for new therapies. We hypothesized that severe AH outcomes are influenced by early specialized care; thus, we examined the impact of time-to-tertiary care (TTTc).

**Methods:**

Adults with cirrhosis or advanced chronic liver disease were enrolled (RH7, NCT04767945). AH was diagnosed using National Institute on Alcohol Abuse and Alcoholism criteria. Primary admission site, TTTc, and adverse outcomes (death or liver transplantation) were analyzed. Patients admitted directly to tertiary care were assigned a TTTc of zero.

**Results:**

Of 221 AD-AH patients, 107 were transferred from secondary care to tertiary care (TTTc >0) and 114 were admitted directly (TTTc = 0). TTTc >0 patients were younger (48.3 vs. 52 years, *P* = .008) and had more severe disease, as shown by model for end-stage liver disease scores (25.5 vs. 20.8, *P* < .001) and Maddrey’s discriminant function (59.3 vs. 40.6, *P* < .001). Propensity-score matching yielded 49 case pairs. The Cox model showed that transfer from secondary care was not associated with increased risk, but delayed transfer (days, hazard ratio = 1.03, 95% confidence interval 1.01–1.05) independently predicted adverse outcomes.

**Conclusions:**

Delayed initiation of specialized care adversely impacts outcomes in AD-AH. If validated, timely care bundles could improve AH survival, similar to sepsis or vascular syndromes.

**Highlights:**

- AD-AH is a common syndrome associated with high short-term mortality.
- There is an unmet need for new prognosis-modifying therapies for AH.
- Currently, in real-life hepatology, refining the existing bundle of care is the only practical option to improve the prognosis of AD-AH.
- Past experience with acute coronary syndromes, stroke, and sepsis, emphasizing symptoms-to-intervention duration, combined with the recent COVID-19 lockdown finding of increased mortality due to skewed access to specialized liver care indicates that focusing on timely specialized care might be key to improved outcome in certain liver conditions.
- In this line, we set out to track the number of days elapsing between admission to SC and referral to TC, coining this interval as “time-to-tertiary care” (TTTc). We examined TTTc as a potential compound surrogate that might influence the prognosis in AD-AH.
- After correcting for important baseline differences, we conclude that the delay of transfer to the tertiary care hospital was independently associated with a worse prognosis with each additional day in TTTc increasing adverse outcomes by nearly 3%.

## Graphical Abstract

**Graphical Abstract:**
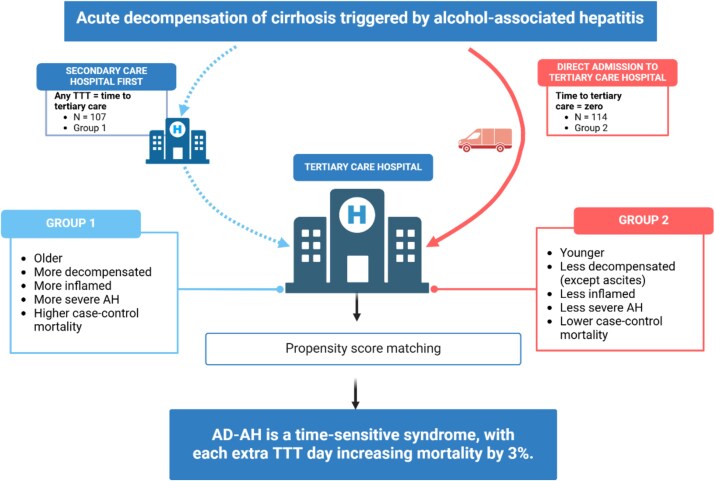

## Teaser

**Short Summary:** This study investigates the impact of time-to-tertiary care (TTTc) on clinical outcomes in patients with severe alcohol-associated hepatitis (SAH). Findings reveal that delays in specialized care are associated with increased mortality, highlighting the importance of timely intervention in managing SAH patients.

## Introduction

According to the Global Burden of Diseases Study Group, there is a high prevalence of decompensated cirrhosis (DC) worldwide (Blachier et al. 2013; Asrani et al. 2019). The most common cause of DC is alcohol-associated liver disease (ALD), which affects up to 70% of patients with advanced chronic liver disease (ACLD) (European Association for the Study of the Liver 2012, NCZI 2019, Szántová 2008, Han et al. 2021, Jepsen and Younossi 2021, Šafčák et al. 2023). The acute phenotype of ALD, alcohol-associated hepatitis (AH), is a distinct clinical syndrome characterized by jaundice and acute liver injury in the setting of heavy alcohol consumption (Arab et al. 2021a, 2021b, 2021c). Severe alcohol-associated hepatitis (SAH) is characterized by features additional to AH: model for end-stage liver disease (MELD) score >20, Maddrey’s discriminant function (MDF) score >31, and mortality rates reaching up to 50% at 90 days (Arab et al. 2021a, 2021b, 2021c, Mathurin et al. 2002, Thursz et al. 2015, Morales-Arráez et al. 2022). Both AH and SAH influence mortality by inducing AD, especially its subtype, acute-on-chronic liver failure (ACLF; Sarin et al. 2019).

At this stage, the lack of effective treatment options beyond corticosteroids (CS) makes SAH difficult to treat. Furthermore, the role of CS is limited to a select group of candidates with SAH, and its prognosis-modifying effect is short-lived and modest at best (Louvet et al. 2009, Thursz et al. 2015). Currently, there are two evidence-based methods of improving the prognosis of patients with AH, both being the emblematic features of tertiary care (TC): (i) timely access and utilization of the existing bundle of care in the so-called window of opportunity and (ii) clinical research of experimental therapeutic options (Degré et al. 2020, Tornai and Szabo 2020, Singal et al. 2021a, 2021b). We first noticed how inaccessibility and delay of bundle of care provided in tertiary setting was associated with increased liver mortality during the COVID-19 pandemic (Tapper and Asrani 2020, Gluckman et al. 2020, Skladany et al. 2021a, 2021b).

We then hypothesized that AH is a time- and setting-sensitive syndrome and the prognosis of AD-AH will be dependent on the time elapsed between the onset of symptoms and initiation of a specialized bundle of care (Skladany et al. 2021a, 2021b, Evans et al. 2021, Jahan et al. 2019). We searched our cirrhosis registry RH7 for surrogate markers of both time from symptoms to care and the level of care, respectively (Arab et al. 2021a, 2021b, 2021c, Tapper and Asrani 2020). We have determined the site of initial care as either secondary care (SC) or TC centers and hypothesized that the outcome may be different and measurable by time-to-tertiary care (TTTc) (Waleed et al. 2020, Singal et al. 2021a, 2021b). Our study aimed to determine if the outcome of patients with AD-AH differs according to the initial setting of liver care (SC vs. TC) and TTTc.

## Materials and methods

After searching our cirrhosis registry RH7 for AD-AH, we explored TTTc as a raw data–derived category and compared the characteristics and outcomes of patients with AD-AH admitted first in SC and later referred to TC (Group 1, any TTTc [TTTc > 0]) with those hospitalized directly in TC (Group 2, zero TTTc [TTTc = 0]) using propensity-score matching. In addition, we explored the impact of TTTc on predicting mortality and/or liver transplantation (LT).

**Figure 1:**
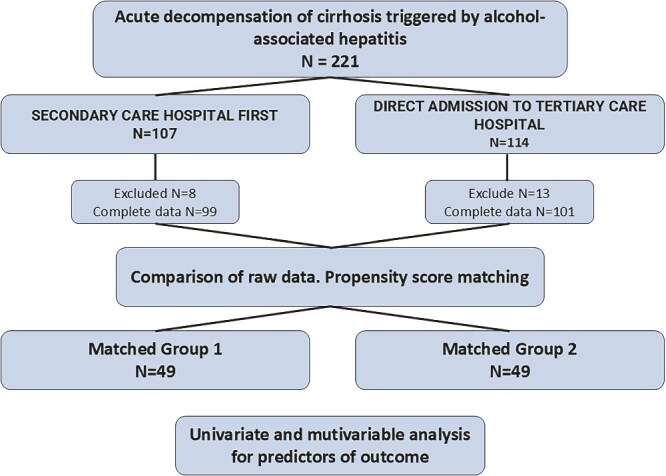
Patient flowchart

The RH7 cirrhosis registry (NCT04767945) at the Department of Hepatology, Gastroenterology, and Liver Transplantation at the University Hospital has been in operation since 2014 as a prospective cohort study (Skladany et al. 2021a, 2021b). In brief, the RH7 contains baseline data (demographics, anthropometry, liver-specific metrics [etiology, acuity of DC, Child–Pugh score, MELD, MDF, ACLF, frailty, etc.]) of adult patients admitted to the liver unit with cirrhosis/ACLD. In the present study, we identified patients hospitalized with AD-AH, with their decompensating event appearing <3 months before their index hospital admission. These patients had available serum bilirubin levels on Days 1 and 7 of hospitalization and at least 1 year of follow-up as of 16 April 2021 (Singal et al. 2018). The diagnosis of AH was made based on the criteria of the National Institute on Alcohol Abuse and Alcoholism (NIAAA) (Crabb et al. 2016). Subsequently, one investigator (D.Ž.) reviewed medical records for evidence of hospitalization in SC before referral to our TC liver unit (Fig. 1). The length of hospital stay in days at the SC was recorded as TTTc. Based on TTTc, we divided the cohort into two groups: Group 1 (any TTTc) included patients hospitalized first in SC, and Group 2 (no TTTc) included patients admitted directly to TC. According to the number of days spent in SC, Group 1 was further divided into five subgroups: 1–5, 6–10, 11–15, 16–20, and >20 days.

SAH was defined as MDF >31 or MELD >20 and ACLF using the EASL-CLIF criteria (European Association for the Study of the Liver 2012). Liver biopsies were only performed on select individuals and indicated based on NIAAA recommendations (not mandatory for diagnosing AH/SAH). We considered patients with SAH ineligible for CS if they presented with contraindications such as gastrointestinal bleeding or uncontrolled infection (Arab et al. 2019). Response to CS was defined by the Lille score at Day 7 (Louvet et al. 2007). For some patients treated with CS in SC, we were not able to retrieve the exact treatment duration. Because the response to CS is an important predictor of outcome, we used a surrogate criterion of “early change” in bilirubin concentration defined as a decrease in the serum bilirubin concentration from the first to the seventh day in TC. This criterion was applied to all patients, regardless of whether they were treated with corticosteroids or not, since data on the duration of CS therapy were not always available (Parker et al. 2021). Acute kidney injury was defined by a recent consensus (Angeli et al. 2015). Hepatic encephalopathy (HE) was diagnosed by an experienced hepatologist based on the clinical evaluation, number connection test, and Stroop test, with evidence of elevated ammonia levels, and was graded using the West Haven classification (Rose et al. 2020). The follow-up continued to at least 1 year after admission to TC or when patients experienced adverse outcomes defined as LT or death. Deaths were verified in the National Registry of Deceased Inhabitants by one investigator.

All procedures involving human participants have been approved and were performed under the ethical standards of the institutional research committee and the 1964 Helsinki Declaration and its later amendments (www.wma.net) or comparable ethical standards. The reported clinical and research activities are consistent with the Principles of the Declaration of Istanbul, as outlined in the Declaration of Istanbul on Organ Trafficking and Transplant Tourism. All patients signed informed consent before enrollment in the RH7, and data acquisition was approved by the local Ethics committee on 21 May 2014.

### Statistical analysis

Results of non-normally distributed variables are displayed as medians and 25th–75th percentiles and were compared using the Mann–Whitney test. Proportions are displayed as numbers and percentages and were compared using the chi-squared test. Differences in numerical parameters and proportions among more than two TTTc groups were assessed by the Kruskal–Wallis test and trend test and with the chi-squared and trend test. Due to substantial baseline differences between the study groups that could markedly confound the effect of TC on the outcome, we carried out propensity-score matching analyses to control for these differences. First, we constructed a backward logistic model to obtain the propensity score—the probability of TC assignment conditional on observed baseline characteristics and confounders for each subject (Austin 2011). The dependent variable was TC, and 16 independent covariates were proposed. To account for potential confounders, we included the following 16 covariates in our propensity-score matching analysis: age, sex, white blood cell (WBC) count, serum albumin concentration, bilirubin concentration on Day 1, early change in bilirubin concentrations, MELD score, MDF, C-reactive protein (CRP) concentration, presence of SAH, overt HE, TTTc, creatinine concentration, relative neutrophil count, presence of ACLF, and LT eligibility. The final model identified six variables independently associated with “direct-to-TC assignment”: SAH, overt HE, WBC count, serum albumin concentration, bilirubin concentration on Day 1, and absence of early change in bilirubin concentrations (area under the receiver operating curve = .839, 95% confidence interval (CI) 0.781–0.887, *P* < .001). Based on the propensity scores, we matched 49 case pairs from the initial cohort of 221 patients allowing a caliper length of 0.2. For the matched cohort, we used the Cox proportional hazard regression to predict death or LT in uni- and multivariate analyses, yielding hazard ratios (HRs) and 95% CIs, and the C-index of the overall accuracy of the model. A significant difference was defined as a *P* value of <.05. Statistical analysis was conducted using the package MedCalc Statistical Software version 2.009 (MedCalc Software Ltd, Ostend, Belgium; www.medcalc.org; 2021) and R (R Foundation for Statistical Computing, Vienna, Austria; https://www.r-project.org/).

## Results

Among the 1109 patients in the RH7 registry, we identified 221 patients with AD-AH who fulfilled the inclusion criteria. There were 107 patients in Group 1 (any TTTc) and 114 patients in Group 2 (zero TTTc). The study flowchart and the results of the initial comparison of the two groups are displayed in Fig. 1 and Table 1, respectively.

**Table 1:** Summary statistics of the cohort of decompensated cirrhosis and alcoholic hepatitis

		**Group 1**	**Group 2**	***P* value**
		***n* = 107**	***n* = 114**	
**Number (%) or median [25th, 75th percentile]**				
Age, years		48.28 [38.53, 56.49]	52.03 [45.05, 61.44]	.008
Sex, *n* (%)	Male	69 (64.5)	68 (59.6)	.490
	Female	38 (35.5)	46 (40.4)	
Hand-grip strength, kg		20.03 [12.00, 31.10]	22.50 [16.63, 30.13]	.096
Severe AH, *n* (%)		101 (94.4)	68 (59.6)	<.001
Steroid ineligible, *n* (%)		13 (12.9)	16 (23.9)	.094
Other complications, *n* (%)	Ascites	20 (18.7)	48 (42.1)	.001
	HE	11 (10.3)	9 (7.9)	
	Jaundice	72 (67.3)	50 (43.9)	
	Bleeding	4 (3.7)	7 (6.1)	
ACLF stage, *n* (%)	No ACLF	36 (33.6)	79 (69.3)	<.001
	Stage 1	34 (31.8)	18 (15.8)	
	Stage 2	25 (23.4)	17 (14.9)	
	Stage 3	12 (11.2)	0 (.0)	
AKI, *n* (%)		32 (29.9)	21 (18.4)	.058
Hepatic encephalopathy, *n* (%)	No HE	35 (32.7)	59 (52.2)	.006
	Stage 1	33 (30.8)	36 (31.9)	
	Stage 2	20 (18.7)	13 (11.5)	
	Stage 3	9 (8.4)	3 (2.7)	
	Stage 4	10 (9.3)	2 (1.8)	
Serum bilirubin, μmol/L	Day 1	348.0 [221.0–491.5]	119.4 [64.3, 273.6]	<.001
INR		1.83 [1.50, 2.37]	1.68 [1.44, 1.97]	.009
Prothrombin time, s		21.49 [18.39, 26.22]	20.17 [17.89, 22.79]	.009
Serum creatinine, μmol/L		81.00 [61.50, 128.50]	65.50 [51.25, 97.25]	.004
Serum albumin, g/L		27.00 [24.00, 3.00]	26.00 [23.00, 31.00]	.828
Maddrey function		59.29 [39.38, 85.54]	40.56 [25.96, 56.73]	<.001
MELD		25.54 [21.22, 31.53]	20.77 [17.13, 25.97]	<.001
C-Reactive protein, mg/L		34.08 [17.74, 67.00]	23.27 [10.71, 42.06]	.001
White blood cells, ×10^9^/L		12.65 [8.12, 17.62]	7.60 [5.73, 11.47]	<.001
Lymphocyte count, ×10^9^/L		1.20 [1.00, 2.05]	1.18 [.80, 2.00]	.189
Neutrophil count relative, %		79.60 [71.25, 86.85]	72.90 [62.35, 79.55]	<.001
Time-to-tertiary care		15.0 [10.5, 24.0]	NA	
Follow-up, days		78.00 [18.00, 784.50]	525.50 [90.50, 1049.50]	<.001

Comparison of groups according to the setting of the initial management: Group 1 (any TTTc), Group 2 (no TTTc). AKI, acute kidney injury; INR, international normalized ratio.

### Comparison of demographic and clinical variables between Group 1 and Group 2

The median age was 48.3 years (interquartile range 3.5–56.5) in Group 1 (any TTTc) and 52 years (45–61.4) in Group 2 (zero TTTc) (*P* = .008). There was no difference between Groups 1 and 2 in the proportion of females (35.5% vs. 40.4%, *P* = .5). Group 1 (any TTTc) had a higher prevalence of SAH (94.4% vs. 59.6%, *P* < .001) and ACLF (69.3% vs. 33.6%, *P* < .001) compared to Group 2. The severity of liver disease and parameters of systemic inflammation were higher in Group 1 (any TTTc) than in Group 2 (MELD: 25.5 vs. 20.8, *P* < .001; MDF: 59.3 vs. 40.6, *P* < .001; WBC count: 12.7 vs. 7.6, *P* < .001; relative neutrophil count: 79.6 vs. 72.9, *P* < .001; CRP concentration: 34.1 vs. 23.3, *P* = .001). The in-hospital and follow-up mortality rates were higher in Group 1 (any TTTc) than in Group 2 (in-hospital mortality 25.2% vs. 13.2%, *P* = .03; mortality at 30 days 35.5% vs. 13.2%, *P* < .001; mortality at 90 days 51.4% vs. 25.4%, *P* < .001).

### Baseline characteristics of Group 1 based on TTTc

**Figure 2:**
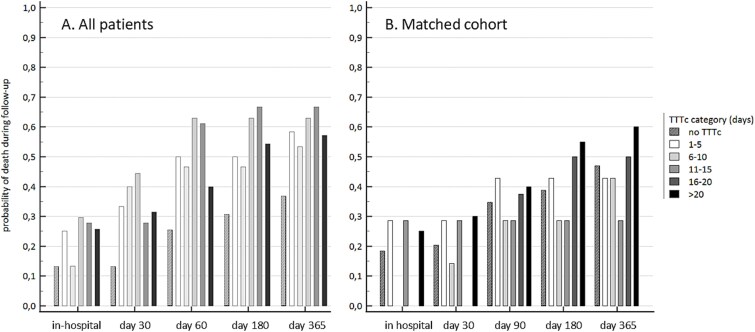
Comparison of mortality during follow-up, no TTTc (direct tertiary care, hatched bars) vs. categories of the time-to-tertiary care (TTTc, solid bars). (**A**) All included patients. Mortality in the hospital: 13.2, 25, 13,3, 29.6, 27.8, 25.7%, *P* = .218; at 30 days: 13.2, 33.3, 40, 44.4, 27.8, 31.4%, *P* = .0004; at 90 days: 25.4, 50, 46.7, 63, 61.1, 40%, *P* = .001; at 180 days: 30.7, 50, 46.7, 63, 66.7, 54.3%, *P* = .003; at 365 days: 36.8, 58.3, 53.3, 63, 66.7, 57.1%, *P* = .03. (**B**) Propensity score–matched patients. Mortality in the hospital: 18.4, 28.6, 0, 28.6, 0, 25%, *P* = .42; at 30 days: 20.4, 28.6, 14.3, 28.6, 0, 30%, *P* = .59; at 90 days: 34.7, 42.9, 28.6, 28.6, 37.5, 40%, *P* = .99; at 180 days: 38.8, 42.9, 28.6, 28.6, 50, 55%, *P* = .724; at 365 days: 46.9, 42.9, 42.9, 28.6, 50, 60%, *P* = .79

In the second part of the analysis, we divided Group 1 (any TTTc) into subgroups based on the number of days spent in SC and compared the impact of TTTc according to baseline parameters on mortality (Table 2). Among the 107 patients in Group 1, the TTTc was 0–5 days for 12 patients, 6–10 days for 15 patients, 11–15 days for 27 patients, 16–20 days for 18 patients, and >20 days for 35 patients. On admission, we observed a statistically significant trend associating TTTc with the bilirubin concentration and MELD score in an inverted U-shaped pattern. We observed the highest bilirubin concentrations in the group with a TTTc of 6–15 days, the highest MELD scores in the group with a TTTc of 1–10 days, and the highest WBC count in the group with a TTTc of 11–20 days. We also observed a significant difference across subgroups in predicting mortality during follow-up (Table 2), with higher TTTc being associated with higher mortality (Fig. 2).

**Table 2:** Comparison of liver-related and inflammatory markers on admission to tertiary care according to a time-to-tertiary care (TTTc) among patients with decompensated cirrhosis and alcoholic hepatitis

	**Group 1 (any TTTc)**					**Group 2 (no TTTc)**		
	**Time-to-tertiary care, days**						***P* value**	
	**1 to 5**	**6 to 10**	**11 to 15**	**16 to 20**	**>20**	**0**	**KW test**	**Trend test**
*n*	12	15	27	18	35	114		
Serum bilirubin, μmol/L								
Day 1	323.9	470.7	467	297.5	256.1	119.4	<.001	<.001
Day 7	309.6	388	398	265	234.8	102.8	<.001	<.001
Day 7 percent change vs. D1	−9.8	−13.4	−7.6	−13.75	−14.9	−11.1	.681	.866
Maddrey function	66.25	63.3	62.3	64.3	49.1	40.6	<.001	<.001
MELD score	25.6	29.2	25.8	26.3	22.9	20.8	<.001	<.001
White blood cells, ×10^9^/L	10.6	11.4	15.4	15.7	11.6	7.6	<.001	<.001
Neutrophils, %	74	84.4	79.2	85	79	72.9	<.001	<.001
C-Reactive protein, mg/L	48.9	31.3	33.9	29.3	34.1	23.3	.04	.003
							Chi-sq test	Trend test
No early change in bilirubin, %	25	20	29.6	33.3	17.1	34.2	.455	.102
Steroid ineligible, %	8.3	13.3	7.4	16.7	14.3	14	.93	.955

KW, Kruskal–Wallis.

### The second step: matched-group analysis

**Figure 3:**
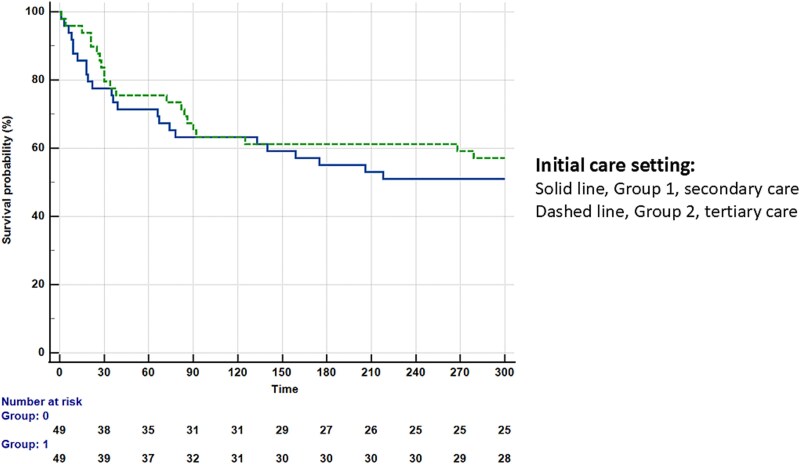
Kaplan–Meier curve comparing the probability of adverse outcomes of patients with decompensated cirrhosis and alcoholic hepatitis according to initial care setting. Solid line: secondary care, dashed line: tertiary care, log-rank test *P* = .997

**Figure 4:**
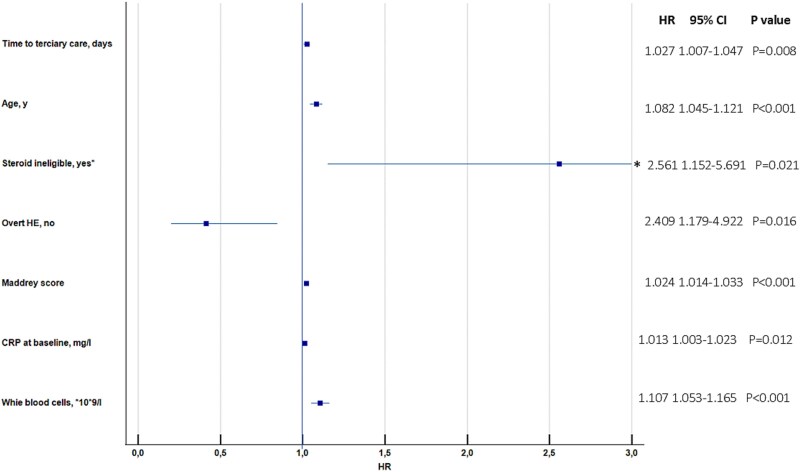
Forest plot of the final Cox model for independent predictors of adverse outcomes among 98 matched hospitalized cases with AD-AH

Propensity-score matching of the two groups yielded 49 case pairs (Table 3). No significant differences were found between matched groups for age, proportion of SAH, cirrhosis complications, serum bilirubin concentration, creatinine concentration, MELD, MDF, or inflammatory markers. In the entire cohort of 98 matched cases, the Cox model showed that the category of SC (Group 1, any TTTc) was not associated with an increased risk of adverse outcomes as displayed in the Kaplan–Meier curve in Fig. 3. However, the model identified seven independent predictors of death or LT: age (HR = 1.08, 95% CI 1.05–1.12), CS ineligibility (HR = 2.56, 95% CI 1.15–5.69), overt HE (HR = 2.41, 95% CI 1.18–4.92), MDF (HR = 1.02, 95% CI 1.01–1.03), WBC count (HR = 1.11, 95% CI 1.05–1.17), CRP concentration (HR = 1.01, 95% CI 1.0–1.02), and numerical time-to-tertiary care (HR = 1.03, 95% CI 1.01–1.05) (Table 4). The results of the model are displayed in the forest plot in Fig. 4. In the group of 49 patients transferred from the secondary care (Group 1, any TTTc), the same model confirmed the independent impact of time-to-tertiary care on adverse outcomes (Table S1).

**Table 3:** Summary statistics and comparison of propensity score–matched groups according to the setting of the initial management: Group 1 (any TTTc), Group 2 (no TTTc)

		**Group 1**	**Group 2**	***P* value**
		***n* = 49**	***n* = 49**	
**Number (%) or median [25th, 75th percentile]**				
Age, years		51.60 [43.52, 59.11]	50.17 [45.08, 57.74]	.974
Sex, *n* (%)	Male	25 (51.0)	30 (61.2)	.416
	Female	24 (49.0)	19 (38.8)	
Severe AH, *n* (%)		43 (87.8)	41 (83.7)	.774
Hand-grip strength, kg		19.20 [12.50, 24.26]	24.73 [16.63, 30.50]	.064
Steroid ineligible, *n* (%)		4 (8.2)	8 (16.3)	.356
Other complications, *n* (%)	HE	5 (10.2)	3 (6.1)	.373
	Ascites	8 (16.3)	15 (30.6)	
	Jaundice	34 (69.4)	30 (61.2)	
	Bleeding	2 (4.1)	1 (2.0)	
ACLF stage, *n* (%)	No ACLF	23 (46.9)	27 (55.1)	.224
	Stage 1	16 (32.7)	10 (20.4)	
	Stage 2	8 (16.3)	12 (24.5)	
	Stage 3	2 (4.1)	0 (0.0)	
AKI, *n* (%)		6 (12.2)	13 (26.5)	.124
Hepatic encephalopathy, *n* (%)	No HE	21 (42.9)	20 (40.8)	.729
	Stage 1	16 (32.7)	20 (40.8)	
	Stage 2	5 (10.2)	5 (10.2)	
	Stage 3	3 (6.1)	3 (6.1)	
	Stage 4	4 (8.2)	1 (2.0)	
Serum bilirubin, μmol/L	Day 1	244.00 [150.40, 348.00]	216.00 [94.30, 394.00]	.977
	Day 7	182.00 [98.00, 302.00]	191.00 [80.30, 355.00]	.994
No early change in bilirubin, *n* (%)		12 (24.5)	12 (24.5)	1.000
INR		1.70 [1.42, 2.07]	1.71 [1.51, 2.23]	.579
Prothrombin time, s	Baseline	20.30 [17.68, 23.63]	20.40 [18.54, 25.02]	.579
Serum creatinine, μmol/L	Baseline	62.00 [57.00, 90.00]	70.00 [51.00, 104.00]	.712
Serum albumin, g/L		27.00 [24.00, 30.00]	26.00 [25.00, 31.00]	.623
Maddrey function		46.34 [28.63, 62.30]	50.28 [35.51, 77.37]	.493
MELD		21.23 [19.58, 25.15]	23.16 [19.74, 29.04]	.346
C-Reactive protein, mg/L		29.26 [14.20, 58.66]	32.69 [19.02, 52.53]	.929
White blood cells, ×10^9^/L		9.60 [6.90, 13.10]	10.50 [6.60, 14.20]	.893
Lymphocyte count, ×10^9^/L		1.50 [1.00, 2.25]	1.20 [.90, 1.92]	.111
Neutrophil count relative, %		75.00 [66.48, 83.00]	77.10 [72.00, 80.00]	.556
Time-to-tertiary care		16.00 [9.00, 26.00]	0.00 [.00, .00]	<.001
Follow-up, days		417.00 [36.00, 1039.00]	337.00 [72.00, 821.00]	.969
Mortality	In-hospital	9 (18.4)	9 (18.4)	1.000
	30 days	11 (22.4)	10 (20.4)	1.000
	90 days	18 (36.7)	17 (34.7)	1.000
	180 days	22 (44.9)	19 (38.8)	.682
	365 days	24 (49.0)	23 (46.9)	1.000

AKI, acute kidney injury; INR, international normalized ratio.

## Discussion

Severe AH is a life-threatening condition with high short-term mortality. In this prospective cohort study, we demonstrated that (i) the clinical profile of an AD-AH patient transferred to TC from SC differs from that of a patient admitted directly to TC in terms of younger age, more severe liver disease, and more inflammation. (ii) After matching the two cohorts, every single day of delay in transferring patients from SC to TC affected the risk of adverse outcome.

This study was conceived based on our experience from the lockdown period, where we interrogated our cirrhosis registry RH7 and found increased non-COVID mortality most probably associated with skewed access to specialized liver care (Kulkarni et al. 2021).

As a consequence, we have conceived of the time-sensitive liver syndromes and set out the current study to first investigate the most probable candidate: AD-AH. Our results lend support to our hypothesis that focusing on the circumstances preceding hospital admission to TC might reveal important prognostic and potential therapeutic targets. Patients entering TC from SC were younger, had more pronounced systemic inflammation, and had more decompensated liver disease—all of which reflect the selection process at SC and opens room for further investigation inside its realm. Another important finding concerning cohort of patients coming to TC from SC was that the follow-up interval after discharge was significantly shorter than in patients from TC. This raises the question whether outcome could be also associated with where the postdischarge follow-up takes place (SC vs. TC). Our findings suggest that outpatient postdischarge management is a crucial part of the bundle of care for AD-AH, encompassing both somatic medicine and psychosocial support with integrated addiction care.

All these points highlight the need for improved communication between TC and SC, initiating research on AD-AH inside SC, issuing SC-tailored guidelines, and promoting integrated addiction medicine within the core bundle of SC care (The ASAM Clinical Practice Guideline 2020).

### AD-AH as an ideal model for testing time and care sensitivity

We have chosen individuals with AD-AH as the optimal group for testing our hypothesis for several reasons: (i) the results from our previous study on liver disease management during the COVID-19 pandemic, where we observed significant challenges in timely access to specialized care and its impact on patient outcomes (Skladany et al. 2021a, 2021b); (ii) high incidence of the syndrome in our region; (iii) its high short-term mortality; and (iv) sensitivity to both the factor of time and factor of healthcare setting proposed by the concept of “window of opportunity” (Degré et al. 2020). In addition, the complexity of AH management, including multidisciplinarity, ACLF (Singal et al. 2021a, 2021b), infections (Louvet et al. 2009; Karakike et al. 2017), malnutrition (Styskel et al. 2019; McClain et al. 2021), addiction (Arab et al. 2021a, 2021b, 2021c), frailty, and early LT (Mathurin et al. 2011), underscores the importance of assessing the timely access to TC covering all these domains. If validated in further studies, the interaction between AD-AH and index hospital admission may have several implications.

First, TTTc may serve as a key target to be optimized with the public-health-scale survival impact. Second, TTTc could shed light on the currently underexplored dynamics of interaction between SC and TC and define prognosis-modifying personalized referral recommendations on the one hand and subsequent personalized outpatient follow-up rules on the other. Third, the influence of TTTc on outcomes in AH may prove significant enough to warrant investigation as an adjunct to existing prognostic models, futility rules, and, importantly, extremely difficult decision-making concerning LT. Last but not least, TTTc might turn out to be condition *sine qua non* for correct interpretation of phosphatidylethanol and other time-dependent biomarkers of alcohol use.

### Acute and follow-up care for AD-AH is the potential therapeutic target

It is challenging to decipher the true effect of TTTc-associated mortality without knowing the numbers, baseline characteristics, and outcomes of patients with AD-AH who were admitted to SC and were never referred to TC. Therefore, there is a need for studies addressing the bundle of care provided in SC (Juniper et al. 2013). Based on the report from the UK on the importance of bundle of care, we have lowered the threshold for SC to refer patients with AD-AH and initiated personal communication and webinars to, among others, advocate for more robust priority pathways which may benefit the most at-risk patients with AH. The report defined the concepts of “missed opportunities” and the “golden window” during the first hours of hospitalization when crucial interventions should have taken place to prevent future adverse outcomes. Data from the report have led to the introduction of a recommended toolkit of care (care bundle) within the first 24 h of hospitalization that is now widely applied in hospitals across the UK (Kiat and O’Mahony 2018). Most catchment SC hospitals in our region do not have qualified hepatologists—ideal intermediates of the process akin to that reported in the National Confidential Enquiry into Patients Outcome and Death. Taken together, our results do lend support to the general notion of the window of opportunity for first-line therapies for AD-AH during which they are maximally effective; in that regard, including TTTc might catalyze optimal outcome in both real-life SC and TC (Sarin and Sharma 2020).

Our study also highlights the importance of quickly identifying nonresponders to CS who would benefit from timely second- and third-line (experimental) therapies that are typically only administered in TC (Arab et al. 2021a, 2021b, 2021c). Furthermore, results from our study suggest that there may be suboptimal utilization of diagnostic tools in SC, such as the Lille model, in identifying individuals who may be candidates for CS therapy (Louvet et al. 2007). For this reason, we resorted to defining a select group of CS nonresponders retrospectively based on an alternative bilirubin criterion, as described earlier (Sarin and Sharma 2020). While SC centers generally provide crucial support, enhancing the integration and systematic approach to addiction care could further improve outcomes for AD-AH patients. As new and promising pharmacotherapies continue to emerge, the timing of treatment initiation is increasingly recognized as crucial. This is true for corticosteroids and other therapies such as interleukin-22, fecal microbial transplantation, and plasmapheresis (Shasthry 2020, Arab et al. 2021a, 2021b, 2021c, Tan et al. 2020). Overall, the findings of this study highlight the urgent need to share with SC the importance of early referral and analyze potential barriers and protocols inside SC. Due attention to the contentious issue of TTTc will necessarily delve deeper and include the intervals prior to SC and even prior to primary care which, apart from their cumulative prognostic value, will open new targetable domains including seeking behavior, stigma and other psychological factors, as well as economic, social, and societal barriers to timely therapy of AH and alcohol use disorder.

Based on our results, we suggest several immediate steps to improve the outcomes in patients with AD-AH: foster promotion of the care bundle including integrated addiction care during the initial hospital stay in SC, coordinate care between SC and TC to streamline pathways for referral, and prospectively evaluate the trafficability of predefined priority pathways of care for the liver diseases most sensitive to a timely referral to TC. We can also focus on the postdischarge management protocol approximation between SC and TC.

**Table 4:** Cox regression model for independent predictors of death or liver transplantation among 98 propensity score–matched patients with decompensated cirrhosis and AH

		**Univariate**			**Multivariate**		
		**HR**	**95% CI**	***P***	**HR**	**95% CI**	***P***
Age, years	*	1.039	0.01–1.068	.007	1.082	1.045–1.121	<.001
Sex, female	*	0.552	0.319–0.954	.033			
Severe AH	*	2.695	0.974–7.453	.056			
Steroid ineligible	*	1.824	0.893–3.725	.099	2.561	1.152–5.691	.021
No early change in bilirubin	*	2.124	1.219–3.7	.008			
ACLF stage (0–3)	*	2.161	1.589–2.939	<.001			
Acute kidney injury	*	2.223	1.225–4.033	.009			
Hepatic encephalopathy, overt	*	3.043	1.682–5.507	.0002	2.409	1.179–4.922	.016
Serum bilirubin, μmol/L							
Day 1		1.002	1.001–1.003	.008			
Day 7		1.003	1.001–1.004	<.001			
% change D1–D7		1.013	1.005–1.02	.001			
Prothrombin time, s		1.096	1.052–1.142	<.001			
INR		2.151	1.531–3.023	<.001			
Serum creatinine, μmol/L		1.006	1.003–1.01	<.001			
Maddrey discriminant function	*	1.019	1.011–1.027	<.001	1.024	1.014–1.033	<.001
MELD score	*	1.087	1.047–1.129	<.001			
C-Reactive protein, mg/L	*	1.011	1.002–1.02	.017	1.013	1.003–1.023	.012
White blood cells, ×10^9^/L	*	1.041	0.999–1.084	.052	1.107	1.053–1.165	<.001
Lymphocyte count, ×10^9^/L		0.853	0.615–1.183	.34			
Neutrophil count relative, %		1.034	1.005–1.063	.02			
Tertiary care only, Group 2	*	1.001	0.595–1.684	.99			
Time-to-tertiary care, days	*	1.01	0.992–1.028	.279	1.027	1.007–1.047	.008
No TTTc vs. any TTTc	*	1.030	0.907–1.169	.65			
Final model C-index					0.79	0.731–0.848	

INR, international normalized ratio.

### Study limitations

There may have been a selection bias in patients referred from SC for patients with more severe disease. This pattern is evident from the differences between the two groups (Table 1). To overcome this limitation, we conducted propensity-score matching. The model achieved an acceptable balance (Table 3) with an area under the receiver operating characteristic curve of .839 (.78–.89) but left 11%–22% of confounding factors unaccounted for. Second, the lack of data on the duration of corticosteroid therapy in SC for some patients with SAH prevented Lille score calculation on these individuals, thereby precluding appropriate analysis of CS nonresponders between SC versus TC. The observed protective effect of a shorter TTTc may thus be explained by the preferential selection of corticosteroid nonresponders for referral to TC. Therefore, we modified the corticosteroid response criteria to include a percentage change in bilirubin concentrations during the first 7 days of TC. While the bilirubin concentrations of corticosteroid responders usually continue to decline beyond 7 days of therapy, those of nonresponders do not (Parker et al. 2021). This approach allowed us to identify spontaneous or corticosteroid-induced responders after 7 days and correct the prognostic model for this important confounder. Interestingly, the rate of change in bilirubin concentrations did not differ among the study groups (before and after matching) or with a further delay before TC. Third, the assessment of the effect of TC delay (i.e. higher TTTc) on the prognosis of patients was inherently confounded by clinical reasoning. This reasoning was reflected in the observed patterns of bilirubin concentration, MELD score, and MDF according to the TTTc in the entire cohort. Despite this limitation, the TTTc was confirmed as an independent predictor of mortality. Finally, our matched data cohort did not have sufficient statistical power to identify the threshold for the critical number of days spent in SC.

## Conclusion

Our study provides new evidence to support the indispensable role of early TC in the management of patients with alcohol-associated hepatitis-triggered acute decompensation (AD-AH) in advanced chronic liver disease. In summary, patients referred to TC from SC were younger and had more severe liver disease, greater systemic inflammation, and higher mortality. In the propensity score–matched cohort, each additional day in SC was associated with a 3% increase in the risk of death. These findings reinforce the importance of timely access to TC and the corresponding bundle of care. Future prospective studies within SC settings are warranted to elucidate the barriers to the identification, treatment, and transferal of these select patients. Furthermore, follow-up studies after TC hospitalization are needed to see long-term outcomes in this population of patients.

## Abbreviations

ACLF, acute-on-chronic liver failure; ACLD, advanced chronic liver disease (real-life equivalent of liver cirrhosis), AD, acute decompensation (of ACLD/cirrhosis); AH, alcohol-associated hepatitis; AD-AH, acute decompensation of cirrhosis triggered by AH; CRP, C-reactive protein; DC, decompensated cirrhosis; HE, hepatic encephalopathy; HR, hazard ratio; LT, liver transplantation; MDF, Maddrey’s discriminant function; MELD, model for end-stage liver disease; RH7, cirrhosis registry of the Division of Hepatology, Gastroenterology and Liver Transplantation; SAH, severe alcohol-associated hepatitis; SC, secondary care; TC, tertiary care; TTTc, time-to-tertiary care; WBC, white blood cell

## Supplementary Material

Table_S1_agae092

## Data Availability

The data that support the findings of this study are available on reasonable request.
