# Supplementary material for: Prospective study on time-to-tertiary care in alcohol-associated hepatitis: space–time coordinates as prognostic tool and therapeutic target
Source: Alcohol Alcohol. 2025 Jan 20;60(2):agae092. doi: 10.1093/alcalc/agae092 (PMC11744045; doi:10.1093/alcalc/agae092)
Supplement: Table_S1_agae092 [file table_s1_agae092.docx]

**Table S1. Cox regression model for independent predictors of death or liver transplantation among 49 patients referred from secondary care (Group 1, any TTTc), sensitivity analysis**

|  | *Univariate* | | | *Multivariate* | | |
| --- | --- | --- | --- | --- | --- | --- |
|  | *HR* | *95% CI* | *P* | *HR* | *95% CI* | *P* |
| Age, years | 1.029 | 0.991-1.069 | 0.132 | 1.099 | 1.045-1.569 | <0.001 |
| Sex, female | 0.705 | 0.332-1.499 | 0.361 |  |  |  |
| Severe AH | 2.168 | 0.512-9.182 | 0.241 |  |  |  |
| Steroid ineligible | 0.887 | 0.210-3.741 | 0.868 |  |  |  |
| No early change in bilirubin | 3.385 | 1.512-7.579 | 0.003 |  |  |  |
| ACLF stage (0-3) | 3.81 | 2.349-6.171 | <0.001 |  |  |  |
| Acute kidney injury | 4.151 | 1.596-10.798 | 0.004 |  |  |  |
| Hepatic encefalopathy, overt | 4.087 | 1.669-10.008 | 0.002 |  |  |  |
| Serum bilirubin, umol/l |  |  |  |  |  |  |
| Day 1 | 1.003 | 1.001-1.005 | 0.012 |  |  |  |
| Day 7 | 1.005 | 1.002-1.007 | <0.001 |  |  |  |
| % change D1-D7 | 1.02 | 1.008-1.031 | <0.001 |  |  |  |
| Prothrombin time, s | 1.097 | 1.047-1.15 | <0.001 |  |  |  |
| INR | 2.118 | 1.444-3.107 | <0.001 |  |  |  |
| Serum creatinine, umol/l | 1.011 | 1.006-1.017 | <0.001 |  |  |  |
| Maddrey discriminant function | 1.021 | 1.011-1.031 | <0.001 | 1.028 | 1.016-1.039 | <0.001 |
| MELD score | 1.146 | 1.079-1.217 | <0.001 |  |  |  |
| C reactive protein, mg/l | 1.01 | 0.998-1.022 | 0.104 | 1.018 | 1.005-1.031 | 0.007 |
| White blood cells, 10*9/l | 1.033 | 0.979-1.091 | 0.234 | 1.103 | 1.031-1.18 | 0.005 |
| Lymphocyte count, 10*9/l | 0.688 | 0.428-1.107 | 0.123 |  |  |  |
| Neutrophil count relative, % | 1.039 | 1.002-1.008 | 0.039 |  |  |  |
| Time to tertiary care, days | 01-Jan | 0.993-1.037 | 0.179 | 1.039 | 1.011-1.067 | 0.006 |
| Final model C-index |  |  |  | 0.794 | 95%CI 0.719-0.868 | |

**TTTc:time to tertiary care, AH:alcoholic hepatitis**
